# Advancing relational methods in agri-food governance research and beyond: Introducing the Socio-Material Actor-Network Analysis (SMANA) methodology

**DOI:** 10.12688/openreseurope.23220.1

**Published:** 2026-04-09

**Authors:** Tanya Zerbian, Violeta Furlan, Daniel López-García

**Affiliations:** 1 Institute of Economics, Geography and Demography, Center of Human and Social Sciences, Madrid, Community of Madrid, 28037, Spain; 2 Basque Center for Climate Change, Bilbao, Basque Country, 48940, Spain

**Keywords:** agri-food governance, social network analysis, participatory network mapping, actor-network theory

## Abstract

To better grasp the messiness of agri-food governance, recent scholarship has called for approaches that foreground its relational, territorial and power-laden character. This paper contributes to this agenda by presenting the Socio-Material Actor-Network Analysis (SMANA) methodology developed in the context of the research project AETERAKIS – A territorialised Agricultural Knowledge and Innovation Systems (AKIS) framework for agroecological transitions.

Building on a hybrid theoretical foundation based on Actor-Network Theory (ANT) and Social Network Analysis (SNA), the SMANA methodology centres its attention on unpacking the agencies, formal and informal interactions, and capacities of different actors in agri-food governance processes, focusing on what infrastructures – physical and non-physical – hold actor relationships together.

Based on its implementation during the AETERAKIS project, the paper proposes the operationalisation of the SMANA methodology through an adaptation of the Net-Map toolkit – a visual-based method for participatory network mapping. This approach enables collective reflection throughout its application and subsequent analysis, supporting local transformation processes. Beyond the immediate purpose of guiding data collection and analysis within the AETERAKIS project, the proposed methodology can assist researchers working on other components or relational aspects of complex, territorial and multi-actor governance processes.

## Plain Language summary

This paper presents a new methodology to better understand how agri-food governance works in practice. Agri-food governance is often complex and messy, shaped by relationships, territories, and power dynamics between different actors. To make sense of this complexity, the paper introduces the Socio-Material Actor-Network Analysis (SMANA) methodology, developed within the AETERAKIS research project.

SMANA combines ideas from Actor-Network Theory and Social Network Analysis. It focuses on how different actors interact, both formally and informally, and on the infrastructures - physical and non-physical - that connect them. These infrastructures help explain how relationships are formed, maintained, and shaped within governance processes.

The paper also explains how SMANA can be applied in practice through an adapted version of the Net-Map tool, a participatory mapping method. This approach allows participants to collectively reflect on their own networks while also generating data for research. Beyond the AETERAKIS project, the methodology can support researchers studying other complex, multi-actor governance processes at the territorial level.

## 1. Introduction

Agri-food governance is a concept of multiple interpretations and uses. As Yap (2023) rightfully points out, a body of literature focuses on specific tools and mechanisms to improve fragmented policymaking, such as policy integration ( Candel, 2014; Parsons, 2022), while others focus on the networks of state and non-state actors for effective participatory policy development and implementation ( Levkoe et al., 2021; Reina-Usuga et al., 2022). However, such focuses have been argued to sideline relevant dynamics of agri-food governance processes, including their historical trajectories, territorial embeddedness, and the unpredictability arising from influences outside the formal boundaries of governance mechanisms ( Moragues-Faus & Battersby, 2021).

Seeking to problematise this understanding, increasing work is bringing forward more relational, territorial, and power-sensitive approaches to the study of agri-food governance. In this view, agri-food governance encompasses the diverse decision-making processes that shape food systems – both what they do and how they work ( Yap, 2023). This conceptualisation directs attention to the decisions and activities of state and non-state actors, each with distinct resources and capacities, that guide, steer, control, or manage food system processes and outcomes through complex, overlapping networks ( Moragues-Faus et al., 2017, Moragues-Faus et al., 2024).

Following this rationale, Social Network Analysis (SNA) is a promising methodology for advancing relational approaches to agri-food governance research. It offers a systematic and quantitative approach to studying relational data, focusing on the positions of actors within networks *(nodes)* and their interconnections *(ties)* ( Borgatti et al., 2022). As a methodological tool, SNA supports the visualisation and measurement of social connections, revealing patterns related to actor interactions that are often difficult to detect ( Light and Moody, 2021). For example, it can shed light on forms of relational power associated with actors’ centrality in multi-actor networks ( Borgatti et al., 2022), as well as on network structures that underpin different modes of governing agri-food systems ( Levkoe et al., 2021).

Nevertheless, SNA presents several challenges for researchers in its implementation. Network data is commonly collected through surveys using either *name generators* – open-ended questions that ask respondents to list the actors with whom they interact – or a *roster format* – a predefined list of actors for respondents to select from. Both approaches, however, involve trade-offs ( Agneessens & Labianca, 2022). Roster-based surveys can be burdensome for participants, particularly when the list is long, and researchers risk overlooking relevant actors who fall outside the predefined boundaries. Name generators, by contrast, rely on participants’ memory and are therefore susceptible to poor recall, which can lead to incomplete network data. More broadly, both methods raise the fundamental issue of network boundary specification – determining which actors should be included and how to ensure that the resulting network adequately reflects the complexity of the system under study ( Laumann et al., 1989).

Alternatively, a performative approach has been proposed to the use of SNA. This approach is usually operationalised through participatory mapping methods using visual, collectively generated data that is then quantitatively analysed through SNA metrics for collective discussion ( Boyle et al., 2021; Hauck et al., 2015; Schröter et al., 2018). Here, SNA’s role is not just about capturing a social phenomenon through a complete or exhaustive network representation. Rather, it is used as a tool for social learning by visibilitsing the cultural, social and institutional bonds that underpin complex governance structures, enabled by visual depictions of that complexity ( Mancilla García & Bodin, 2020; Scott, 2015). However, it is precisely this complexity that makes relations difficult to trace and actors challenging to identify ( Boyle et al., 2021).

Governance dynamics are messy, evolving within shifting actor constellations, uneven power relations, and context-dependent interactions over time ( Forney et al., 2025). Simplified SNA visuals do not necessarily depict this complexity ( Rosas & Knight, 2019). Greater analytical attention has therefore been directed towards adopting relational approaches that move beyond social dynamics alone to include socio-material elements, thereby providing entry points for understanding complex governance systems ( Forney et al., 2025), such as that proposed by Actor-Network Theory (ANT) ( Latour, 2005) or assemblage theory ( DeLanda, 2016). This focus emphasises the instruments, artefacts, and procedures through which governance is enacted, as well as the diverse interactions between human and non-human actors that hold it together. However, the operationalisation of such an approach remains challenging in empirical research, with specific methodologies and methods not necessarily outlined. At the same time, relational approaches, such as ANT, have been argued to pay insufficient attention to power asymmetries and social inequalities within networks ( Elder-Vass, 2008), while also suffering issues of boundary-setting ( Spies & Alff, 2020), and, thus, operationalisation.

This paper seeks to advance the implementation of a relational approach to analysing governance dynamics by leveraging the complementary strengths of ANT and SNA and, in doing so, potentially surpass their limitations. For this purpose, it proposes a hybrid methodology that integrates Actor–Network Theory (ANT) with a performative approach to SNA, which we have called Socio-Material Actor-Network Analysis (SMANA). Developed within the AETERAKIS project – *A territorialised Agricultural Knowledge and Innovation Systems (AKIS) framework for agroecological transitions*, this innovative methodology aims to capture relational dynamics bringing into attention the socio-material elements that hold these relations togethe. Through this approach, it seeks to produce collective social learning while improving relationship tracing and actor identification to analysing social networks.

### 1.1 Overview of the AETERAKIS project

The proposed methodology is part of the AETERAKIS project, conducted between January 2025 and July 2027 and funded as a Marie Skłodowska-Curie Postdoctoral Fellowship 2023. The project focuses on the territorial organisation and the role of AKIS in supporting agroecological transitions, with the aim of developing the AETERAKIS framework ^1^ ( https://cordis.europa.eu/project/id/101146631). It combines participatory network mapping methods under the hybrid SMANA methodology across four provincial Spanish case studies – Barcelona, València, Araba, and Madrid. The project explores how formal AKIS infrastructures and governance for organic farming interact with agroecology-oriented farmers’ personal knowledge networks, and how these interactions shape the orientation, circulation, and use of knowledge and innovation for agroecological transitions.

The focus on formal AKIS infrastructures for organic farming stems from the assumption that, as an institutionalised production model ( Moschitz et al., 2021; Ewert et al., 2023), organic farming might have already established AKIS infrastructures that could support deeper agroecological transitions. While we acknowledge the tensions and limitations associated with this assumption (see, for example, Migliorini and Wezel 2017; Lampkin et al. 2020), discussing them falls beyond the scope of this paper, which primarily concerns the proposal of the SMANA methodology and its operationalisation. In this paper, AKIS are used as an exemplary empirical case to illustrate how the proposed methodology provides a structured entry point for tracing actors, socio-material interactions, and power-laden relations in complex and hard-to-bound governance systems, while also facilitating collective reflection and deliberation.

Examining AKIS governance and dynamics is particularly suitable for this methodological reflection. AKIS exemplify the relational, multi-actor, and multi-level complexity through which agri-food governance processes unfold. In the European context, AKIS refers to a dynamic and open system of actors, institutions, and networks involved in the creation, sharing, application, and transformation of knowledge and innovation in agriculture and related fields ( EU SCAR, 2019). It concerns farmers, advisors, researchers, public authorities, educational institutions, consumers, and other actors, including private companies such as input suppliers and other agrifood chain actors ( Charatsari et al., 2023). Notably, AKISs constitute a multi-actor, multi-level system in which learning and innovation emerge through interaction among a diversity of actors and collective action ( EU SCAR, 2012). Thus, their nature is not static but continuously *in the making* ( Knierim et al., 2017; Klerkx et al., 2012). Beyond actors and their relationships, AKIS also includes the broader socio-political environment – the “rules of the game”- comprising the norms, values, and institutional arrangements and decision-making processes that shape how actors operate ( Charatsari et al., 2024; Fieldsend, 2020).

## 2. Combining ANT with SNA: Introducing the SMANA methodology

SNA and ANT approaches are among the most prominent frameworks for analysing networks, yet they stem from distinct epistemological and ontological traditions ( Vicsek et al., 2016). ANT advances a flat ontology within a realist and (new) materialist paradigm. It rejects a-priori hierarchies of interactions and binary distinctions, allowing *both human and non-human actors* to conform networks, with agency, understood as relational and emerging from the interrelations in which actors are enrolled ( Latour, 2005; Gamble et al., 2019). By contrast, SNA is commonly situated within a broader positivist paradigm ( Vicsek et al., 2016). It emphasises the measurement and representation of social relations and interaction patterns within an observed system or community composed mainly of *human* actors. Although these approaches are often considered incompatible, the SMANA methodology aims to bring ANT and SNA into productive dialogue to overcome some limitations of each approach discussed in the introduction.

A key premise of ANT is its focus on how heterogeneous associations between human and non-human actors are formed, and how these associations are stabilised and maintained over time. It poses particular attention to the practices and devices through which collectives of actor-networks are provisionally assembled ( Vicsek et al., 2016). In Latour’s (2005) words *“we have to take non-humans into account only as long as they are rendered commensurable with social ties and also to accept, an instant later, their fundamental incommensurability”* (p. 78). This means that non-human actors are analytically considered insofar as they become relevant within the associations that constitute actor-networks. They are rendered commensurable only in terms of the roles they play in mediating these associations – through which they are also transformed or even conformed, not because they are necessarily equivalent to social actors or social ties. Such commensurability is therefore situational and ever-changing; not finished nor characterised by any essence beyond such situated interrelations.

In this line, a key role in actor-networks is that of non-humans in the form of *mediators.* Mediators do not simply transmit action or information without modification. Instead, they transform, translate, redirect or amplify relations as associations are formed. Their effects cannot be inferred from their inputs alone, as they actively shape how actors relate to one another and have a crucial role in stabilising actor-networks, thus having, in terms of Latour (2005), their own agency. The concept of mediators is particularly helpful in providing a starting point to trace the social structures of governance systems. Durable social connections and structures are not only based on human means. They are fragile and ephemeral if they do not have something that sustains them across time and space ( Latour 2005; Forney et al. 2025). By shifting action onto material, technical or organisational elements, mediators give relations durability and inertia, allowing power, coordination and governance arrangements to extend beyond transient interactions ( DeLanda, 2016). Looking at the role of non-humans in this way is also the key in bringing ANT and SNA together, as it could allow researchers to follow the connections that stabilise governance systems.

Similar to ANT, the underpinning theoretical assumption of SNA is that, to explain social phenomena, attention must be given to their inherent relationality. Indeed, networks in SNA need to be understood by including dyadic variables (attributes of pairs of actors and characteristics of relations among actors) ( Borgatti et al., 2022; Vicsek et al., 2016). Although in SNA those considered actors are predominantly individuals or social groups, such as institutions or organisations, the notion of *two-mode networks* can set a bridge with ANT’s idea of mediators in actor-networks. Two-mode networks (also known as affiliation networks) are composed of two distinct sets of nodes (actors of mode I and actors of mode II) – commonly individuals and the events, organisations, or institutions to which they are connected ( Borgatti et al., 2022). Here, the focus is on how nodes of mode I are connected to one another through their affiliation with or participation in nodes of mode II. Direct connections among actors within the same mode are not represented, unless the network is transformed into a one-mode projection derived from these shared affiliations (ibid.). In this sense, mode II nodes can be understood as playing a mediating role within the network, as they enable and structure relations among mode I nodes, thereby providing a conceptual bridge between both theories.

Building on this insight, the proposed hybrid SMANA methodology analyses two-mode affiliation networks within the context of complex agri-food governance; in this paper exemplified through AKIS. Rather than treating organisations, events or institutions as mode II nodes, as is common in conventional SNA, the SMANA methodology foregrounds socio-material infrastructures as heterogeneous mediators of governance systems. These infrastructures are understood as the *means* – particularly concerning physical or non-physical spaces, such as marketing spaces, test farms, platforms, research or R + D projects, online platforms for information sharing, dissemination events, through which AKIS actors develop, exchange, transfer and implement knowledge and innovation. To maintain coherence with ANT while avoiding a strictly positivist interpretation of network data, the SMANA methodology adopts a performative approach to SNA, as explained in the introduction. This approach retains formalised network representations and quantitative analyses, while using them as tools to trace associations and elicit collective reflection rather than as definitive descriptions of a given system. In doing so, it leverages the strengths of both ANT and SNA, combining selected elements of both. The following section illustrates how the SMANA methodology has been implemented in the context of AETERAKIS project.

## 3. Methods: Operationalising the SMANA methodology through visual data collection and analysis methods

The SMANA methodology is operationalised by using an adaptation of the Net-Map method ( Schiffer & Hauck, 2010; Schiffer & Waale, 2008). The Net-Map method aims to understand complex actor-networks, power relations, and influence dynamics within a governance or decision-making context using SNA and a visual, interactive data collection approach. Originally developed for individual interviews, Net-Map has been increasingly applied in group settings and collaborative planning, allowing for the visualisation of shared knowledge and the co-construction of system representations ( Hauck et al., 2015; Boyle et al., 2021). Accordingly, through visual and participatory network mapping, Net-Map supports participants in identifying leverage points and limitations in navigating complex systems to achieve common goals. In the case of AETERAKIS project, the Net-Map method was deployed through one-day workshops for participatory network mapping.

The implementation of the Net-Map method is usually guided by a specific research question, such as “Who influences XY policy development or XY system achievement of sustainability goals” ( Hauck et al., 2015). This question is put into practice via either workshops or interviews through the following objectives: (1) determining what actors are relevant in achieving/influencing system goals; (2) eliciting their level of influence and alignment with these goals, and (3) identifying the linkages that are currently present to determine whether increased strategic networking can improve in the achievement on these goals ( Schiffer & Hauck, 2010). As a visual-based method, it draws heavily on material objects for eliciting answers and facilitating discussion. Small figurines and actor cards are utilised to represent actors that are positioned by participants on large sheets of paper, checker pieces or similar round disks that are stacked to build influence towers below or next to the actors, and color pens to draw actor links. As this requires active participation, the method prompts participants to stay focused on the objectives of the workshop ( Gava et al., 2025; Hauck et al., 2015; Schröter et al., 2018).

**Figure 1.**
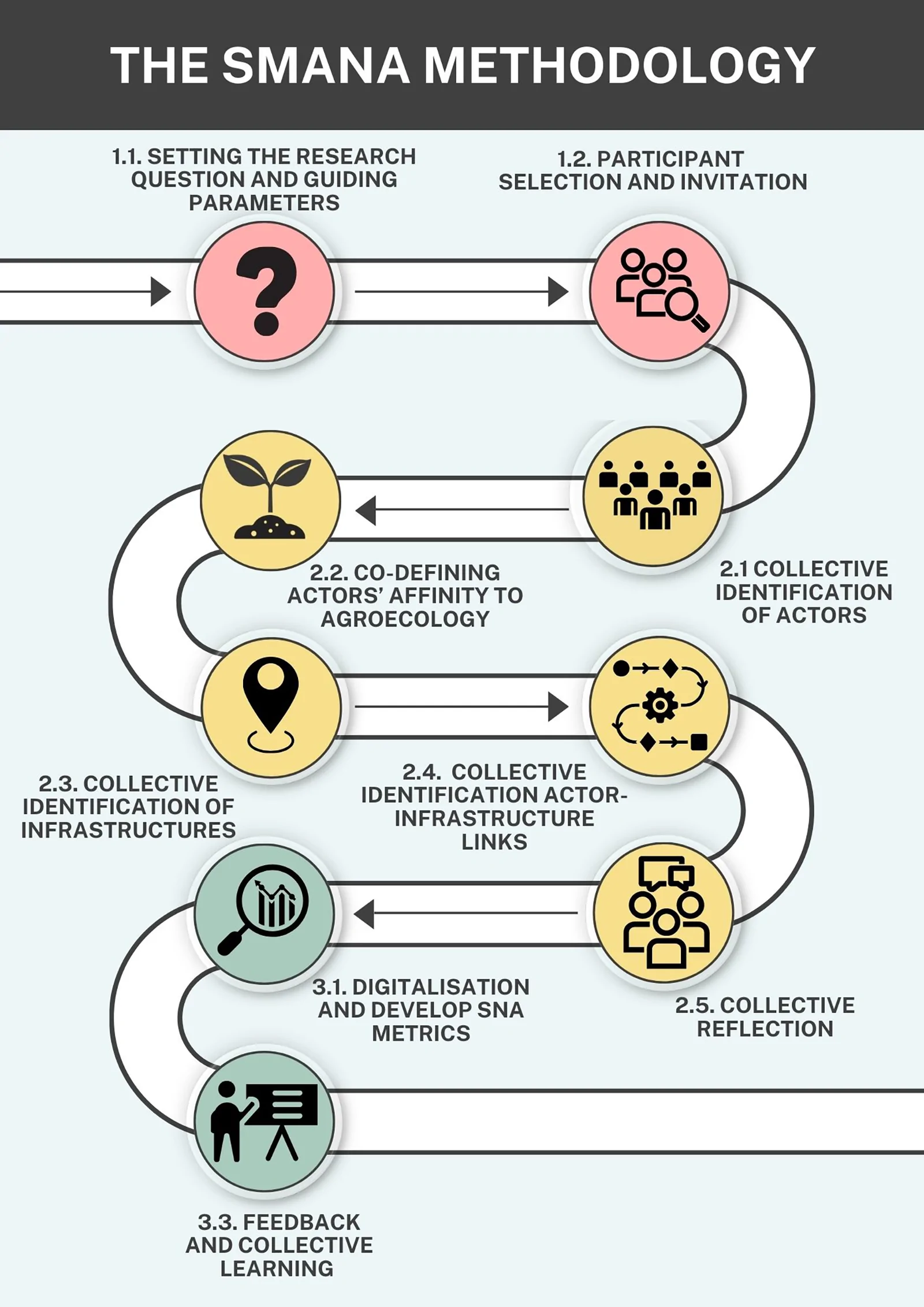
Step-by-step guide SMANA methodology. Pink circles refer to steps of Phase 1 (Preparation, pretesting and sampling), yellow circles to steps of Phase 2 (Conducting the workshops), and green circles to steps of Phase 3 (Analysis).

We adapted this method to include the identification of the mediators of actor interactions, following the proposed SMANA methodology. To do so, besides figurines for actors (people, organisations), figures for mediating socio-material infrastructures are also utilised, with links then drawn between actors-infrastructures. Besides this adaptation, the elicitation of influence was removed from the exercise. As Boyle et al. (2021) indicate, this step could lead to power conflicts when performing workshop activities, particularly related to state and non-state actors. Although this is a relevant aspect, the use of the SMANA methodology during the AETERAKIS project prioritised facilitating local transformation processes through an improved understanding of AKIS governance. The potential for generating conflicts, both within and beyond the workshops, was therefore carefully planned. Moreover, influence and power relations can be measured using quantitative SNA metrics ( Borgatti et al., 2022). Conversely, actors’ alignment with agroecological transitions was indeed assessed, following the original Net-Map approach of eliciting alignment with a system’s desired goals. Figure 1 provides a step-by-step guide to implement the SMANA methodology through the adapted Net-Map method.

The next sections develop this step-by-step guide further, and discuss the different stages of the implementation of the SMANA methodology, including which SNA metrics to use for this purpose as deployed within the AETERAKIS project.

## 4. Use cases: Implementation of the SMANA methodology in the AETERAKIS project

### 4.1 Preparation, pretesting and sampling

Despite the performative use of SNA, such as through the Net-Map method, partly reducing the need to define strict network boundaries, clear guiding parameters are still required ( Schiffer & Hauck, 2010). These include specifying what is being studied and identifying the actors or actor categories likely to be present in the system, drawing on prior experience or existing literature. It also requires careful consideration of participant selection, as the resulting network reflects the perspectives, positions, and degrees of engagement of those invited ( Hauck et al., 2015). In this regard, selection should explicitly aim to capture a diversity of perspectives, based on actors’ roles in the system and their viewpoints on the system under analysis. Moreover, the number of workshop participants needs to also be considered. As in the case of other group-based data collection methods ( Stewart et al., 2007), 6–8 participants was considered a good number to facilitate fluid group discussion and reflection.

Given the action-oriented approach of the AETERAKIS project, participant selection was guided by their relevance to agroecological transitions in the territory. Moreover, the selection followed the 8 recommendations for the design of participatory processes in environmental management developed by Del Vente et al. (2016), since its accuracy allowed it to adapt it to the research context. We took into account, number of participants, gender, age, ways of knowing, and power asymmetries to align participation with inclusiveness and equity principles. Specifically, participants were selected based on their in-depth knowledge of the AKIS infrastructure for organic agriculture and related models and/or their active involvement in agroecological initiatives. The aim was to ensure a balance between critical reflection and grounded insight into existing systems of knowledge exchange, transfer, and co-creation for organic farming from an agroecological perspective. Moreover, participant selection also focused on including perspectives of a variety of fields of expertise (ways of knowing) within the different AKIS-subsystems, including public administrations, farming advisory services, education and research, farmers, and the agri-food supply chain sector and civil society. The intention was to include at least one representative from each sub-system.

With the starting hypothesis that AKIS for organic farming could serve as a stepping-stone toward agroecological transitions, the one-day workshops in each case study sought to achieve three core objectives: (1) to map the formal AKIS infrastructure in the territory, including actors and infrastructures, for organic farming as perceived by participants; (2) to identify its limitations and potential leverage points of this system in supporting agroecological transitions; and (3) to generate recommendations for policy, action, and intervention based on collective reflection of the resulting map.

As noted above, clarifying terminology is essential to ensure the robustness of the method, particularly with regards to meeting its objectives. Accordingly, the workshop protocol was pretested amongst the authors. The last version of the workshop protocol utilised in the AETERAKIS project after this process is available in the official DIGITAL.CSIC repository (see http://hdl.handle.net/10261/419174 - Zerbian, 2026a).

During the pre-testing phase, it was identified that clear working definitions of the concepts knowledge, innovation, actors, and infrastructures were needed, particularly regarding the differentiation between an actor and an infrastructure. The following working definitions were proposed:

• *Knowledge* as a learned, experiential, or evidence-based understanding of a topic used for decision-making;
• *Innovation* as a collective process of improving practices, organisation, or commercialisation through knowledge exchange, not limited to technology;
• *Actors* as organisations or entities involved in generating, exchanging, or applying knowledge and innovation within the territory including the categories of: advisory services, research, farmers and farmer-based organisations, education, public authorities, the food supply chain (upstream and downstream, including consumers and input suppliers), and non-governmental organisations (NGOs) ( Charatsari et al., 2023; EU SCAR, 2012; Fieldsend, 2020; Knierim et al., 2015, 2017);
• *Infrastructures* as physical or non-physical spaces, project, platform, etc. that enable - and thus intermediate - diverse interactions and connectivity among actors.

Moreover, actor-infrastructure-actor links were distinguished between information flows, resource/material flows, and planning, following previous SNA studies analysing agri-food governance dynamics ( Levkoe et al., 2021; Zerbian et al., 2022). Similar to the original Net-Map method, the materials utilised, and thus prepared beforehand, included (with exceptions of the exclusion of the checker pieces and inclusion of figures for infrastructures):

1. Large sheets of paper to draw the resulting AKIS maps.
2. Post-its to write down actor and infrastructures’ names.
3. Coloured 3D cylinders representing different actors based on previously defined actor AKIS categories.
4. Coloured 3D cubes representing the infrastructures, differentiating between physical and non-physical ones based on colour.
5. Different coloured felt pens to draw different types of links.

The testing phase also made clear the need for collaborative facilitation of the workshops. Given their complexity, joint facilitation was essential to set an environment of collaboration among actors that have diverging backgrounds, experiences and knowledge types. Collaborative facilitation also helped participants stay focused on the task, manage the different dynamics unfolding in parallel, and ensure the smooth progression of the sessions. At the same time, it was considered that working collaboratively allowed the facilitators to support one another, distribute attention and responsibilities, and maintain the quality and consistency of the process throughout the workshop. Following the five pillars proposed by Albert-Fonseca et al. (2025) for transformative research allowed the research team to generate caring spaces for themselves and the participants during workshops for knowledge co-creation.

### 4.2 Conducting the workshops

The participatory workshops followed a sequenced structure combining individual and collective reflection that integrated four steps.

**Figure 2.**
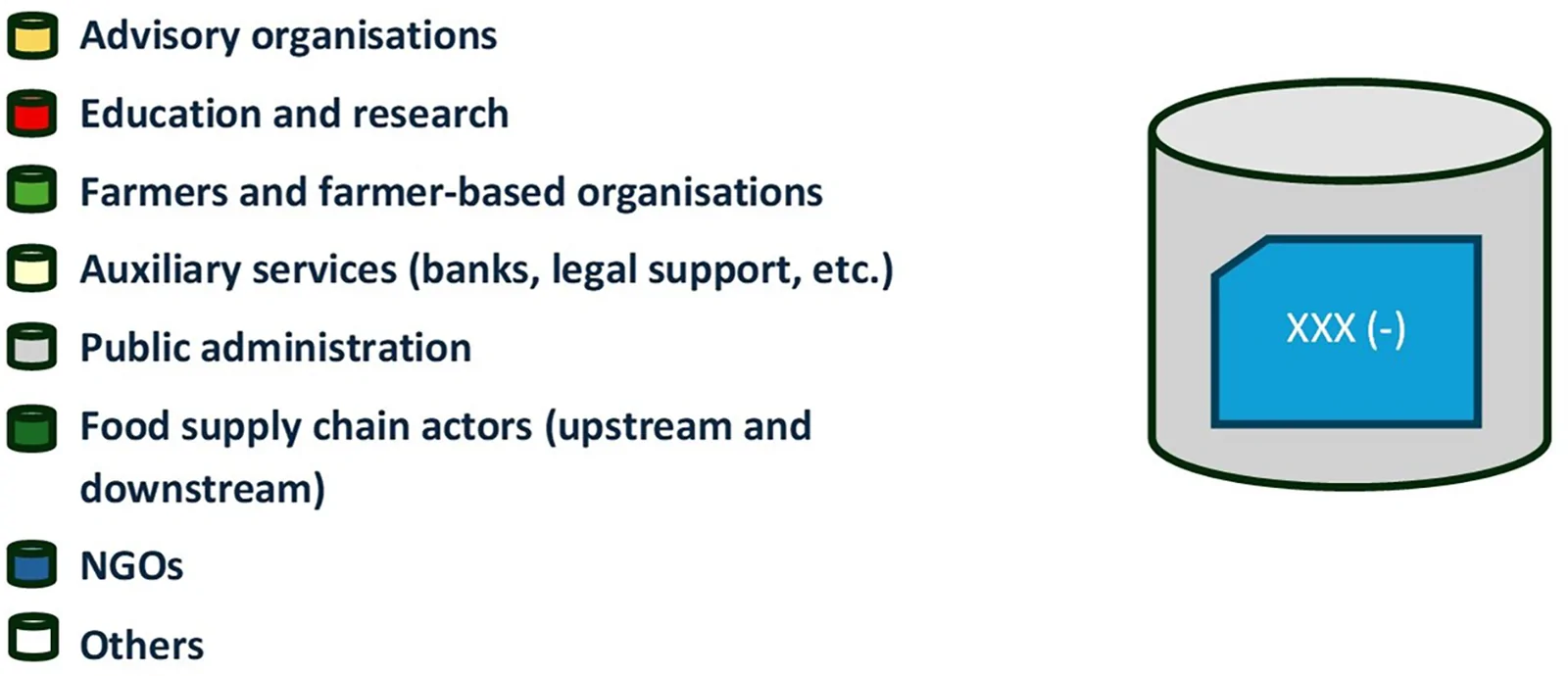
Colour-based actor categorisation and example of an identified AKIS actor. Post-it note colours were used randomly and did not convey any analytical meaning. Within the post-its participants wrote the name of the actor, agroecology alignment (+/=/−), and indicated whether there were individuals that either facilitated or limited agroecology-related actions.

**The first step** involved participants identifying relevant actors of the AKIS for organic farming within the territory of each case-study and assessing their alignment with agroecological principles. Actors were represented using coloured figurines (cylinders) - see Figure 2 - based on each of the actor categories developed in the preparatory phase.

While these categories served as a guide, previous research highlights that the boundaries within and between AKIS subsystems are often blurry, and hybrid profiles and overlapping mandates are common. For instance, advisory services may be delivered by actors embedded within commercial firms, cooperatives, or NGOs, sometimes combining public and private funding streams ( Knierim et al., 2015). Therefore, actor categories were not treated as rigid, with the main function of the actor within AKIS as its categorisation but recognising the hybridity of actors and overlapping mandates during workshop discussions. At the same time, actors outside the administrative boundaries of the territories could also be considered as long as they have an active role in the AKIS dynamics of the analysed territory. In line with the AKIS definition explained before, the key inclusion criterion was that actors be engaged – directly or indirectly – in generating, sharing, or using knowledge and innovation for organic farming and related fields of the selected territories. This served as the main criterion for delimiting the boundary of the network during the workshops. While identifying relevant actors, participants were also asked to elicit **whether these actors were aligned with agroecology** – yes (+), no (−) or having a neutral stance (=), which was indicated in the post-its with actor names.

**Figure 3.**
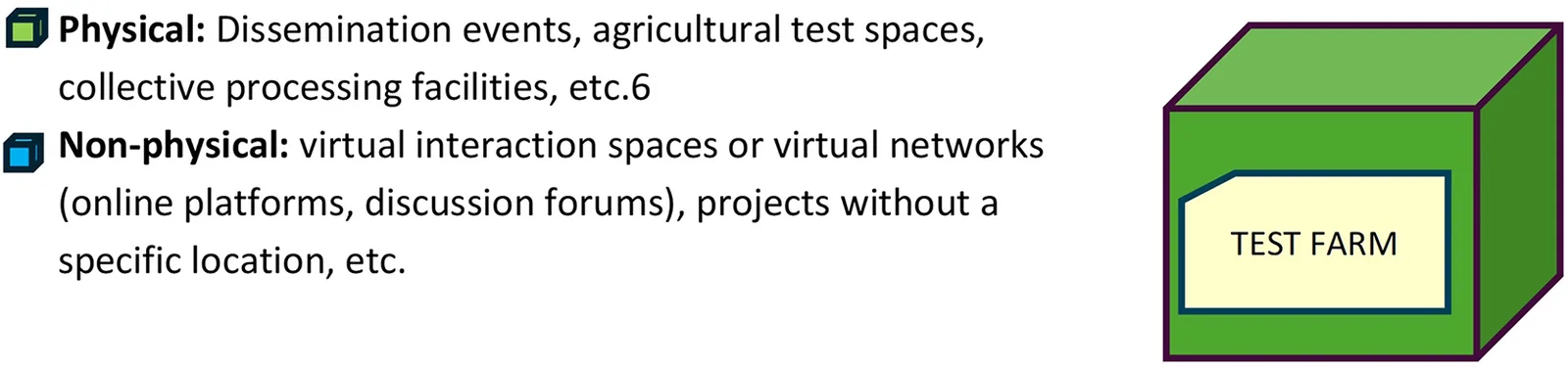
Colour-based infrastructure categorisation and example. Post-it note colours were used randomly and did not convey any analytical meaning.

In **step 2**, participants subsequently mapped mediation infrastructures – physical or non-physical – where knowledge exchange, coordination, and co-creation occur among the identified actors ( Figure 3). These included training events, working groups, collaborative projects, online platforms, among others. Participants were explicitly prompted to discuss the nature and purpose of each infrastructure, including whether it served to transfer information, coordinate activities, support decision-making, or foster experimentation. This dialogue helped to clarify the functional roles of these spaces in current AKIS dynamics and their relevance to agroecological transitions.

**Figure 4.**
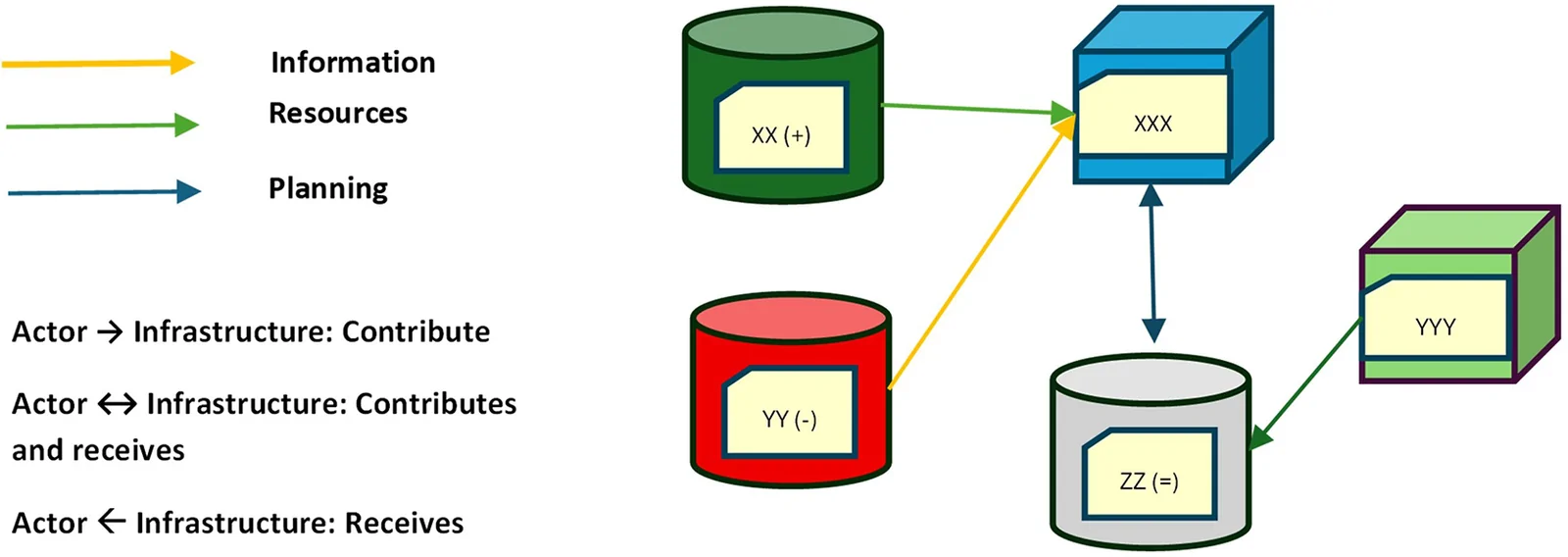
Example of link tracing between actors-infrastructures-actors with directionality and link categorisation. Each colour represents a different link – yellow for information, green for resources, and blue for planning. Links also had three different options for directionality – receiving, contributing, and contributing and receiving.

Next, for **step 3**, participants collectively identified which actors were involved in which infrastructures. Actors were then physically positioned close to the infrastructures in which they actively participated in the large sheets of paper. This exercise helped to make actor–infrastructure relationships immediately visible. Finally, links were drawn between actors and infrastructures, and between actors who shared the same infrastructures, utilising colour-coded markers and indicating the direction of the links, distinguishing between information flows, resource/material flows, and planning, as explained earlier (see Figure 4).

The **final step** of the workshop focused on group discussion, interpretation, and strategic reflection. Participants analysed the mapped AKIS network structure, identifying central or missing actors, underused or critical infrastructures, and potential barriers to agroecological transitions such as silos, asymmetries of power based on resource or planning flows, weak cross-sectoral collaboration, and misalignment of relevant actors with agroecological principles. The sessions closed with collective identification of actionable leverage points – actors, relationships, and infrastructures that could be strengthened or reoriented to better support agroecological transitions, along with discussion of funding flows, policy and institutional support needed to do so.

### 4.3 Analysis

As explained by Hauck et al. (2015), analysing Net-Map data can take many forms depending on the research objectives. A common practice, beyond collectively analysing the network visually during the workshop (as in the final stage of the SMANA methodology), is to translate the information generated into SNA metrics and share these results with participants for a further round of collective reflection. This process was also conducted in the AETERAKIS case studies, with the digitised networks fed back to the participants through an online meeting for further discussion and reflection.

Hand-drawn network maps were converted into an edgelist, a standard data structure for SNA in which each row represents a tie between two nodes and can include link attributes ( Borgatti et al., 2022), such as link type or directionality in this case. Ties connected actors to the identified AKIS knowledge and support infrastructures. Two-mode networks require analytical approaches that differ from those used for one-mode networks. Available options include analysing each mode independently using metrics specifically developed for two-mode networks, projecting the two-mode network into one-mode networks and applying conventional SNA measures, or analysing the two-mode structure directly ( Murphy & Knapp, 2017). The SMANA methodology focuses on the third approach, given the relevance of identifying the role of infrastructures as mediators between actor interactions.

Metrics specialised for two-mode networks were calculated using xUCINET (V. 0.0.2.0020) in RStudio. Network visualisations were produced using the *sna* (V. 2.7–2) and *igraph* (V. 2.0.1.1), packages. For SNA analyses, all data were treated as undirected to allow for better two-mode data analysis ( Borgatti et al., 2022). This was then accompanied by a qualitative thematic analysis ( Braun & Clarke, 2006) of the transcriptions of the workshops and feedback meetings to gain more insights into the *why* and *how* of the observed network structures and metrics. To analyse the relational dynamics of the AKIS governance of the cases, including the role of infrastructures in mediating interactions among actors, we focused on a set of complementary SNA measures addressing both node relevance and overall network structure. Specifically, we examined degree centrality, network density, community detection, and core–periphery structure.

• Degree centrality was used to assess the relevance of both actors and infrastructures within the AKIS. In two-mode networks, degree centrality corresponds to the number of connections a node has to nodes in the opposite mode. For actors, this reflects engagement with knowledge and support infrastructures; for infrastructures, it indicates the number of actors connected to them and their functional reach within the AKIS.
• Network density was used as an indicator of overall structural cohesion and was calculated as the proportion of observed actor–infrastructure ties relative to the maximum number of possible ties between the two modes.
• To identify substructures within the governance network, community detection and core–periphery analysis were applied using a dual-projection approach ( Everett & Borgatti, 2013). The Dual-projection approach projects the information of both one-mode projections (actor–actor and infrastructure–infrastructure), losing less information when used in algorithms and models ( Borgatti et al., 2022).
  ○ Community detection identified clusters of actors engaging with similar infrastructures and groups of infrastructures mobilised by similar sets of actors, capturing patterns of coordination and functional differentiation.
  ○ Core–periphery analysis identifies highly popular and densely interconnected actors and infrastructures. Actors can be part of the core given their active participation in infrastructures, and infrastructures given the amount of actors that participate in them, indicating the concentration of governance and coordination functions.

SNA metrics were integrated with the previously defined actor categories and with a refined categorisation of infrastructures from the thematic analysis of workshop data. Qualitative analysis was used to interpret the structural positions identified through SNA by clarifying the functions of key actors and infrastructures within AKIS dynamics. In particular, it allowed us to explain how different infrastructures operate as spaces for interaction, the purposes they serve, the types of knowledge and resource flows they promote, and how these relate to central, core, or peripheral positions, as well as to thematic groupings identified through community detection.

## 5. Discussion: Lessons learned from the application of the SMANA methodology

The implementation of the SMANA methodology in the case studies of the AETERAKIS project, as described before, offers relevant insights into its strengths and limitations. We describe next the lessons learned from this process, pointing to relevant aspects to be further developed in future implementations or adaptations of the methodology.

First, implementing the methodology through participatory network mapping offered multiple points of discussion of the quality and stability of AKIS actor relationships. Significantly, approaching networks through infrastructures helped reveal contrasts between stable, institutionalised patterns of interaction and more informal dynamics, where coordination relied on ad hoc arrangements and personal ties. For instance, physical spaces functioned as recurrent points of coordination and planning, whereas time-bound projects were primarily understood as funding channels with constrained deliberative capacity, shaped by their limited duration and predefined objectives. Moreover, being able to lay out the different types of interactions – information, resources, and planning, as well as their directionality, elicited discussions regarding who exchanges information with whom, who provides funding, and what kinds of participation are promoted and whether these are mutual or one-sided.

One limitation was the difficulty to differentiate clearly between actors and infrastructures for participants. Projects, platforms, and organisations frequently overlapped, raising the question of what constitutes an “infrastructure” in the network: whether it should be understood as an actor in its own right, as a mediator among actors, or as a temporary assemblage tied to specific funding or objectives. This ambiguity became particularly evident where long-running projects or shared platforms were described both as places of interaction and as collective actors with agency. Yet, as Vicsek et al. (2016) rightly note, ANT captures this complexity by allowing individuals or organisations to be treated as actors, while also understanding them, when viewed at a different scale, as actor-networks in themselves. From this perspective, the observed blurriness should not be read as a methodological shortcoming, but rather as an empirical reflection of the layered and relational nature of agri-food governance processes and AKIS dynamics, in which roles, mediations, and forms of agency are continuously reconfigured. Indeed, discussing these forms of blurriness during the workshops proved useful in eliciting the relevance of certain actors in enacting different roles within AKIS dynamics. This reinforces the value of the SMANA methodology and its implementation by adopting a performative SNA approach for creating spaces for collective sense-making rather than categorical closure.

Differentiating between types of infrastructures was relevant to support in-depth discussion on their role in configuring AKIS governance dynamics. However, this was a challenge to fully capture during workshops. Projects, online platforms, advisory initiatives, and physical meeting spaces were all mapped, yet the adapted Net-Map method offered limited analytical leverage for examining how these infrastructures condition interaction in different ways. The maps made mediation visible, but not always its quality or mode, other than by the types and directionality of the links drawn.

These challenges raise questions about whether the SMANA methodology requires more than just punctual workshops to reach its full potential. Indeed, the technique provided largely static network representations, leaving temporal and historical dimensions underexplored. This was reflected in participants’ difficulty in refraining from drawing direct linkages between actors, as discussions repeatedly gravitated towards actor–actor exchanges configurations, rather than actor-to-infrastructure relations. Participants noted that relations currently represented as direct exchanges were often shaped by earlier periods in which interaction was mediated by specific projects or spaces. What appears as a stable tie in the present drawn networks frequently rested on a shared historicity of collaboration. This suggests that repeating the same mapping exercise at different moments could help capture how mediation and interactions evolve to sediment into more durable relationships. Notably, a deeper discussion on the types of mediators and the implications of their differential mediation roles could be a relevant topic for further workshops or discussions.

Concurrently, it is important to note that, in practical terms, the successful implementation of the methodology depends on facilitators having specific training in facilitation and participatory methods, as recommended more broadly for participatory research ( Scher et al., 2023). Such training is essential for navigating conflict and unequal influence, guiding participants towards active and respectful engagement in group discussions and the mapping process, given the workload and intensity of the workshops. This is particularly relevant when feeding back results to participants. In one of the cases of the AETERAKIS project, the presentation of the SNA metrics, identifying who occupies central positions and who does not, generated discomfort and critical reactions among some participants, particularly as actors they considered central to the governance dynamics did not appear as such in the resulting map.

For this reason, careful consideration of participant recruitment is required to ensure a coherent diversity of perspectives in the mapping process, including adaptation of time schedule to enhance participation. For example, in the case of Araba, participation barriers were especially evident for women facing work overload, a challenge rooted less in the technique itself than in the broader context of women’s role in agroecological transitions ( Vizuete et al., 2024). Simultaneously, it is also extremely important to make clear to participants that the primary aim of the methodology is not to produce a complete or objective representation, but rather to elicit collective reflection through the process of mapping and its later analysis through SNA. As Boyle et al. (2021) explain, performative SNA requires sensitivity to processes of information exclusion, balanced against the objectives and scope of the research, with the question of ‘who participates’ then also becoming a central analytical concern in the collective interpretation of the resulting maps. Such an approach also brings SMANA closer to what is understood as ‘performative’ new materialisms ( Gamble et al., 2019). For example, in the case studies of the ATERAKIS project, participants stressed the importance of explicitly asking who is not included as a reflexive practice.

Despite these challenges, participants identified several advantages of the SMANA methodology. The participatory workshops prompted reflection on how to improve collaboration among actors who rarely work together due to institutional, sectoral, or resource constraints, such as farmers and researchers. The capacity of the approach to induce collective reflection and learning was particularly evident during feedback meetings, where SNA metrics, analyses, and multiple visualisations of the digitised networks were presented. These sessions helped clarify the distribution of funding, work, and information, as well as how AKIS governance is segmented into subgroups with distinct thematic orientations and limited interaction. Discussing these patterns enabled participants to identify infrastructures and actors that function either as enablers or as sinks for agroecological transition, while also identifying the need for new connections that may support future collaboration and innovation. By combining participatory mapping with collective interpretation of network metrics and visualisations, the process supported knowledge co-production for local transformation processes and stimulated social learning. SMANA ultimately has the potential to generate new synergies and associative forms within the analysed territories, by supporting the co-creation of shared imaginaries on complex socio-material interrelations.

## 6. Conclusion

This paper set out to advance relational approaches to the study of agri-food governance by proposing and empirically testing SMANA, a hybrid methodology integrating ANT with a performative use of SNA developed under the AETERAKIS project for the case of AKIS. Grounded in participatory network mapping and collective interpretation, SMANA was developed to better capture the messiness, territorial embeddedness, and socio-material mediation that characterise complex governance dynamics, while also supporting social learning and knowledge co-production. Rather than treating networks as fixed structures, the methodology foregrounds how relations are enacted, stabilised, and transformed through socio-material arrangements, while simultaneously enabling the generation of network data suitable for SNA.

The application of SMANA demonstrated its value in bridging ANT’s sensitivity to the role of mediation of non-human actors to surface relational complexity, with SNA’s capacity for systematic analysis and visualisation of relational patterns. At the same time, it highlights methodological challenges related to temporality, power asymmetries, participant selection, and the limits of static representations. These challenges should not be understood as shortcoming, but as inherent tensions in operationalising relational methodologies in complex governance contexts. Rather than seeking exhaustive or objective representations, SMANA is best understood as a reflexive and flexible framework for operationalising relational approaches to complex, multi-actor governance processes, fostering collective reflection, knowledge co-production, and social learning. Accordingly, its relevance could lie not only in the specific context of agri-food systems or AKIS, but also in its applicability to complex governance processes more broadly.

## Ethics and consent

This study was reviewed and approved by the Ethics Committee of the Spanish National Research Council (Comité de Ética del Consejo Superior de Investigaciones Científicas, CSIC) (Reference No. [166/2025]). All procedures involving human participants were conducted in accordance with the ethical standards of the CSIC.

Following these standards, written informed consent was obtained from all participants prior to their participation in the study. No identifiable personal details or images of participants are included in this publication.

## Use of Generative AI tools

The authors used ChatGPT (OpenAI) version 5.2 to improve the clarity, grammar, and overall readability of the manuscript. The tool was used exclusively for language editing and stylistic refinement. All conceptual development, methodological design, analysis, and interpretation of results are the authors’ own.

## Data Availability

DIGITAL.CSIC repository. AETERAKIS workshop protocol. URI: http://hdl.handle.net/10261/419174 DOI: https://doi.org/10.20350/digitalCSIC/18110 ( Zerbian, 2026b). This project contains the following underlying data:
• 20260217_Supplementary Material I_SMANA.pdf Data is available under the terms of the CC BY 4.0 license.

## Footnotes

^1^
The AETERAKIS framework will introduce a conceptualisation of territorialised AKIS and identify key characteristics and relational dynamics through which AKIS can be reconfigured to better support agroecological transitions, including patterns of actor diversity, knowledge circulation, governance arrangements, and cross-scale linkages.
