# Supplementary material for: Advancing relational methods in agri-food governance research and beyond: Introducing the Socio-Material Actor-Network Analysis (SMANA) methodology
Source: Open Res Eur. 2026 Sep 12;6:95. Originally published 2026 Apr 9. [Version 2] doi: 10.12688/openreseurope.23220.2 (PMC13583356; doi:10.12688/openreseurope.23220.2)
Supplement: Peer review history

# Reviewer report: Reviewer response for version 2

**DOI:** 10.21956/openreseurope.27020.r80926

**Published:** 2026-09-16

**Authors:** Isidro Maya-Jariego

**Affiliations:** 1 Department of Social Psychology, Universidad de Sevilla (Ringgold ID: 16778), Sevilla, Andalusia, Spain

The authors have responded to the reviewers' comments by incorporating appropriate changes.

Are sufficient details provided to allow replication of the method development and its use by others?

Partly

Is the description of the method technically sound?

Yes

Are the conclusions about the method and its performance adequately supported by the findings presented in the article?

Partly

Is the rationale for developing the new method (or application) clearly explained

Yes

If any results are presented, are all the source data underlying the results available to ensure full reproducibility?

Partly

Reviewer Expertise:

Social psychology; Network analysis; Community intervention.

I confirm that I have read this submission and believe that I have an appropriate level of expertise to confirm that it is of an acceptable scientific standard.

# Reviewer report: Reviewer response for version 1

**DOI:** 10.21956/openreseurope.25124.r73099

**Published:** 2026-05-20

**Authors:** Patricia Manzano Fischer

**Affiliations:** 1 Department of Food Sciences, Universidad Autonoma Metropolitana - Lerma (Ringgold ID: 414538), Lerma, State of Mexico, Mexico

The manuscript presents the SMANA methodology, a hybrid approach that combines ANT and SNA to study complex agri-food governance systems, particularly the Agricultural Knowledge Innovation System (AKIS). The objective of this methodology is to overcome limitations of both approaches by integrating ANT´s attention to socio-material mediation and non -human actors with SNA´s capacity for structural and quantitative network analysis. SMANA uses an adapted version of the Net-Map method, where participants collaboratively map actors, infrastructure, and their relationships during participatory workshops.

 The study applies the methodology within the AETERAKIS project to analyse governance dynamics related to agroecological transitions. The findings show that focusing on infrastructure as mediators helped participants identify how knowledge exchange, coordination, funding and planning processes are organised within AKIS. The integration of participatory mapping promoted social learning and identification of leverage points for improved collaboration. 

 The study identified methodological challenges and limitations. 

 The manuscript presents a valuable methodological contribution and is well structured overall. SMANA provides a flexible framework for analysing socio-material governance networks, and the study case is clearly presented. The manuscript requires only minor revisions.

 Areas where the manuscript could be strengthened include:

 1.- Clarifying whether the main innovation lies in the use of infrastructures as mediators, the performative interpretation of SNA, the adaptation of Net-Map, or if it is the integration of all these elements.

 2.- The manuscript acknowledges the ambiguity between mediators and infrastructure. Readers would benefit from clearer criteria to help understand when something is treated as an infrastructure, as an actor or as both.

 3.- Clearly defining the concept of performative SNA, as it remains somewhat underdefined in this context.

 4.- Thematic Analysis is mentioned; however, no details are provided. It would be helpful to explain how the information from this analysis was integrated with the SNA results and how it contributed to the interpretation of findings.

Are sufficient details provided to allow replication of the method development and its use by others?

Yes

Is the description of the method technically sound?

Yes

Are the conclusions about the method and its performance adequately supported by the findings presented in the article?

Yes

Is the rationale for developing the new method (or application) clearly explained

Yes

If any results are presented, are all the source data underlying the results available to ensure full reproducibility?

Yes

Reviewer Expertise:

Food system sustainability and governance

I confirm that I have read this submission and believe that I have an appropriate level of expertise to confirm that it is of an acceptable scientific standard, however I have significant reservations, as outlined above.

# Response

**Published:** 2026-08-13

**Authors:** Tanya Zerbian

**Affiliations:** Instituto de Economia y Geografia y Demografia, Madrid, Community of Madrid, Spain

Thank you for the positive feedback and identifying the manuscript as a relevant contribution. Below please find our responses to your comments and the changes made in the manuscript.

*We have clarified that the innovation of the paper lies in the integration of the three elements identified. The central contribution is the use of an SNA analytical approach to operationalise an ANT-informed understanding of agri-food governance particularly through the use of infrastructures as mediators. This is enabled by a performative interpretation of SNA, understood not only as a tool for representing networks, but also as a way of making actors, relations, and mediating infrastructures visible and open to collective discussion. The adaptation of Net-Map serves this purpose methodologically, providing a participatory procedure through which relational data can be generated, visualised, and analysed. Thus, the innovation lies in combining ANT, a participatory – and thus –  performative use of SNA, and an adapted Net-Map approach into a coherent methodological strategy for analysing infrastructures as relational mediators.*

*We have included this in the first paragraph of the conclusion.* *We have addressed the point regarding the ambiguity of actors and infrastructures further in the discussion (Section 5), where we now reflect on how this distinction has been refined after the discussions raised during the workshop and bringing this in closer conversation with ANT. We also indicate these categories should be seen as contingent, functional and relational rather than fixed or mutually exclusive ones, complying with the theoretical underpinning of the methodology.* *We have better explained the performative use of SNA in the Introduction. In the revised manuscript, we have reorganised the section where it was explained and provided a clearer definition of performative SNA, specifying that its performative role lies not in producing a complete representation of a network, but in making certain actors, relations, and governance problems visible, discussable, and actionable through collective reflexivity.* *We have also refined Section 4.3 to clarify our analytical strategy and have added a final paragraph to explain how thematic analysis contributed to the interpretation of findings.*

# Reviewer report: Reviewer response for version 1

**DOI:** 10.21956/openreseurope.25124.r73104

**Published:** 2026-05-14

**Authors:** A Amarender Reddy

**Affiliations:** 1 ICAR-National Institute of Biotic Stress Management (NIBSM), Raipur, India

The manuscript presents an innovative and timely methodological contribution by integrating Actor-Network Theory and Social Network Analysis into the proposed SMANA framework. The paper is conceptually strong, well-structured, and highly relevant for analysing complex agri-food governance systems and AKIS dynamics. The participatory adaptation of the Net-Map approach and the emphasis on socio-material mediation are particularly valuable.

 However, the paper would benefit from greater clarity on the operational distinction between “actors” and “infrastructures,” as this ambiguity appears repeatedly during implementation. The paper is too lengthy, but relevant sub-sections are missing. Like what are the specific characteristics of “actor” and “infrastructure”.  The graphical presentation is well, but the ultimate illustration of integrating and depicting all in one graphical form is missing, it may be incorporated. Authors also should explicitly mention how one can draw lessons, while preparing the maps. Further discussion on handling temporality, power asymmetries, and validation of network representations would strengthen the methodology, although they have mentioned it in the limitations Some sections are overly detailed and could be condensed for readability. Minor language editing (See “elements that hold these relations togethe.”) is also recommended to improve flow and remove repetition.

 Authors may take some real examples to explain like Farmers Producer companies (FPCs) etc. Rani, C. R., Reddy, A. A., & Mohan, G. (2023). From formation to transformation of FPOs. Econ Polit Wkly, 58(36), 15.

 Overall, this is a significant methodological contribution with strong potential applicability beyond agri-food governance, but minor revisions are needed to improve conceptual clarity and presentation.

 The manuscript presents an innovative and timely methodological contribution by integrating Actor-Network Theory and Social Network Analysis into the proposed SMANA framework. The paper is conceptually strong, well-structured, and highly relevant for analysing complex agri-food governance systems and AKIS dynamics. The participatory adaptation of the Net-Map approach and the emphasis on socio-material mediation are particularly valuable.

 However, the paper would benefit from greater clarity on the operational distinction between “actors” and “infrastructures,” as this ambiguity appears repeatedly during implementation. The paper is too lengthy, but relevant sub-sections are missing. Like what are the specific characteristics of “actor” and “infrastructure”.  The graphical presentation is well, but the ultimate illustration of integrating and depicting all in one graphical form is missing, it may be incorporated. Authors also should explicitly mention how one can draw lessons, while preparing the maps. Further discussion on handling temporality, power asymmetries, and validation of network representations would strengthen the methodology, although they have mentioned it in the limitations Some sections are overly detailed and could be condensed for readability. Minor language editing (See “elements that hold these relations togethe.”) is also recommended to improve flow and remove repetition.

 Authors may take some real examples to explain like Farmers Producer companies (FPCs) etc. Rani, C. R., Reddy, A. A., & Mohan, G. (2023). From formation to transformation of FPOs. Econ Polit Wkly, 58(36), 15.

 Overall, this is a significant methodological contribution with strong potential applicability beyond agri-food governance, but minor revisions are needed to improve conceptual clarity and presentation.

Are sufficient details provided to allow replication of the method development and its use by others?

Yes

Is the description of the method technically sound?

Yes

Are the conclusions about the method and its performance adequately supported by the findings presented in the article?

Yes

Is the rationale for developing the new method (or application) clearly explained

Yes

If any results are presented, are all the source data underlying the results available to ensure full reproducibility?

Yes

Reviewer Expertise:

agricultural economics, institutional economics, governance

I confirm that I have read this submission and believe that I have an appropriate level of expertise to confirm that it is of an acceptable scientific standard, however I have significant reservations, as outlined above.

# Response

**Published:** 2026-08-13

**Authors:** Tanya Zerbian

**Affiliations:** Instituto de Economia y Geografia y Demografia, Madrid, Community of Madrid, Spain

*We thank the reviewer for the positive feedback and view of the article as a significant contribution, as well as for the points for improvement. Below please find our responses and the changes we have made in the revised version.*

*We have included more information on the operational distinction between "actors" and "infrastructures" in Section 4.1. However, as rightfully pointed out by the reviewer, the operational distinction between “actors” and “infrastructures” became especially relevant during the implementation of the mapping process. We have therefore addressed this point in the discussion (Section 5), where we now reflect on how this distinction builds from the theoretical underpinning of the methodology, particularly the performative understanding of SNA as a process that does not simply represent networks, but also supports knowledge co-production, collective interpretation, and reflection on how relations are configured.*

*Drawing on the discussions raised during the workshops, we use these insights to refine the definitions of actors and infrastructures, while clarifying that they should be understood as functional and relational categories rather than fixed or mutually exclusive ones, complying with the ANT approach.* *Regarding adding a graphical representation depicting all in one, we believe that Figure 4 already depicts how a potential mapped network could look, bringing together the different elements discussed in the manuscript in a single graphical form. We have clarified this more explicitly in the revised text and are willing to revise the figure if needed.* *In response to the comments, we have further explained in the methodology how lessons can be drawn during the map-making process through guiding questions. *   Further discussion on handling temporality, power asymmetries, and validation of network representations would strengthen the methodology, although they have mentioned it in the limitations. *We have also included more detail on handling temporality, power asymmetries, and validation of network representations in* Section 4 and Section 5, also responding to Reviewer 1.  Where possible, sections have been reduced. Language editing has also been conducted.   We have also included real examples, such as that suggested, to discuss the role of infrastructures.

# Reviewer report: Reviewer response for version 1

**DOI:** 10.21956/openreseurope.25124.r72195

**Published:** 2026-04-16

**Authors:** Isidro Maya-Jariego

**Affiliations:** 1 Department of Social Psychology, Universidad de Sevilla (Ringgold ID: 16778), Sevilla, Andalusia, Spain

The text proposes a methodology for analyzing agri-food governance networks. It is clear and well-structured, reviewing relevant and current literature. Network analysis can contribute to a relational and territorial understanding of agri-food governance, particularly regarding the relative centrality (and power) of different actors and the identification of relevant subgroups in decision-making.

 The text suggests integrating Actor-Network Theory with network analysis. In theory, networks that combine actors and objects (that is, "human and non-human actors") can be operationalized as Mode 2 networks and are therefore amenable to empirical analysis. However, it is worth noting that network analysis and actor-network theory often stem from different epistemological foundations. Although two theories can be integrated methodologically, this does not necessarily imply that such integration is possible or coherent at the epistemological level, where irreconcilable ontological assumptions and validity criteria may persist. It would be worthwhile to reflect on the limitations of the methodological proposal suggested here. It is noted that "these approaches are often considered incompatible", but that a "productive dialogue" between them is proposed. As presented, it appears that the analytical potential of network analysis (e.g., obtaining indicators of actors' intermediation capacity) is actually being used to serve actor-network theory. In other words, one approach takes precedence over the other.

 The case study is relevant and well-presented. Furthermore, the implications of relational processes for the governance of the agri-food sector are adequately justified.

 Some of the challenges involved in using name generators, name lists, or the network boundary specification problem are identified. Reference is made to the "performative use" of network analysis, although the source of this term is not indicated, among the different uses of networks in intervention. It should be noted that the use of the term "performative" in the network interventions literature may have a different meaning or implications than its use in the context of actor-network theory.

 Methodologically, it would also be useful to distinguish between data collection through strictly analytical procedures (for example, asking each independent actor to systematically evaluate their relationships with each of the other identified actors) and through consensus-based evaluation procedures (for example, the qualitative assessment of a group of actors gathered in a focus group). The second approach is likely better suited to what the authors intend to do regarding agri-food governance. In this second case, the proposal could benefit from applying the principles of validity in qualitative research (see, for example, Small and Calarco, 2022). (Ref 1)

 To illustrate the methodology, the case study is presented in sufficient detail, with a focus on the lessons learned, which may be useful for other researchers who wish to use the same approach. It may be of interest to compare this with other studies that have also been inspired by the Net-Map technique, such as Mihăilă et al. (2026). (Ref 2)

 The combination of centrality indicators (e.g., degree), structural cohesion (e.g., density), and subgroups (e.g., community detection) covers some of the key dimensions of network variability. For comparative purposes, it would possibly be useful to incorporate an assessment of fragmentation or the number of components in the analyzed networks.

 It is noted that "physical spaces functioned as recurrent points of coordination and planning." This is an interesting observation. However, it should be noted that interaction patterns sometimes overlap with common places or settings. This is the case with the concept of "behavior settings," where it is not possible to separate interactions from the places where they occur. One version of this analytical strategy would be to identify relationship patterns associated with specific places or community contexts.

 I find the observation that the application of this technique, with its strong qualitative basis, depends heavily on the facilitators' skills particularly pertinent.

 Overall, the text provides valuable insights into the experience of applying participatory-relational techniques to describe agri-food governance. I believe the section on the technique's limitations could be expanded, explicitly outlining the cautionary notes regarding what it cannot contribute.

 Minor corrections: visibilitsing = visibilising.

Are sufficient details provided to allow replication of the method development and its use by others?

Partly

Is the description of the method technically sound?

Yes

Are the conclusions about the method and its performance adequately supported by the findings presented in the article?

Partly

Is the rationale for developing the new method (or application) clearly explained

Yes

If any results are presented, are all the source data underlying the results available to ensure full reproducibility?

Partly

Reviewer Expertise:

Social psychology; Network analysis; Community intervention.

I confirm that I have read this submission and believe that I have an appropriate level of expertise to confirm that it is of an acceptable scientific standard, however I have significant reservations, as outlined above.

## References

[1] : Qualitative Literacy.2022; 10.2307/j.ctv2vr9c4x 10.2307/j.ctv2vr9c4x

[2] : From stakeholder mapping to statistical modeling: an illustrative demonstration of end-to-end Net-Map methodology for health governance analysis. *Archives of Public Health* .2026; 10.1186/s13690-026-01879-2 10.1186/s13690-026-01879-2 PMC1310777041845516

[3] : Types of network-based interventions: a conceptual clarification. *Connections* .2025;46(1) : 10.2478/connections-2025-000215-24 10.2478/connections-2025-0002

# Response

**Published:** 2026-08-13

**Authors:** Tanya Zerbian

**Affiliations:** Instituto de Economia y Geografia y Demografia, Madrid, Community of Madrid, Spain

We thank the reviewer for the careful revision of the manuscript and the extremely relevant methodolgical and conceptually relevant comments. Below are our responses: We agree that the manuscript needed to more explicitly address the methodological and epistemological tensions involved in combining ANT and SNA. We have therefore clarified this issue in the Introduction and in Section 2, specifying that our proposal does not aim to fully reconcile ANT and SNA at the epistemological level. Rather, we use SNA as a methodological support for an ANT-informed, relational, and performative reading of networks. We have also added a final paragraph to make this contribution explicit. Regarding the claritifcation of the performative use of SNA, we had already included a description of this idea in the Introduction, but we recognise that the paragraph was not sufficiently clear or well structured. In the revised manuscript, we have reorganised this section and provided a clearer definition of performative SNA, specifying that its performative role lies not in producing a complete representation of a network, but as a tool for making certain actors, relations, and governance problems visible, discussable, and actionable.

 We have also clarified in the introduction to Section 3 that our proposal is based on a consensus-based evaluation procedure, as implemented in the workshops, rather than on independent actor-by-actor assessments of all possible relationships. We particularly thank the reviewer for pointing us to Small and Calarco (2022). Rather than incorporating their principles as a formal checklist in the manuscript, we have used them throughout Section 5 to discuss the lessons learned from the application of SMANA and its limitations, as they helped us engage more critically with the methodology’s application. Regarding comparing the lessons learnt of the application of the proposed methodology, we had already compared our approach with other Net-Map-inspired studies, particularly in the limitations section through the discussion of Boyle et al. (2021) and Schröter et al. (2018a/b). In response to the reviewer’s comment, we have expanded this comparison by incorporating Mihăilă et al.’s (2026) recent study. *To respond to comments regarding extensions or including more analytical layers to the methodology, we have included these acomments in now Section 5.3 as potential adaptations of the methodology, such as including other metrics (fragmentation or the number of components).* *With regards to the point that interaction patterns may overlap with common places or settings is highly relevant for mobilising an ANT-informed perspective to analyse agri-food governance dynamics. Indeed, similar to the concept of “behaviour settings”, ANT explicitly advances the notion that the materiality of the spaces in which relations occur are part of an ‘assemblage’ of what makes social relations. However, this implication had not been explicitly developed in the discussion or in relation to potential analytical extensions. We have therefore added this point to the revised manuscript (Section 5.2), noting that the proposed set of SNA metrics could be complemented by an analytical layer examining how specific relationship patterns are associated with particular physical spaces given their materiality.*
